# Single nucleotide polymorphisms in the ANGPTL4 gene and the SNP-SNP interactions on the risk of atherosclerotic Ischaemic stroke

**DOI:** 10.1186/s12883-021-02138-3

**Published:** 2021-03-09

**Authors:** Chaoxiong Shen, Daofeng Fan, Huajun Fu, Chong Zheng, Yinjuan Chen, Zhizhou Hu

**Affiliations:** Neurology Department, Longyan First Hospital Affiliated to Fujian Medical University, No. 105, Jiuyi North Road, Longyan, 364000 Fujian China

**Keywords:** Single nucleotide polymorphisms, Gene, ANGPTL, Atherosclerotic ischemic stroke

## Abstract

**Objectives:**

The purpose of this study was to investigate the impact of single nucleotide polymorphisms (SNPs) in the ANGPTL4 gene and the SNP–SNP interactions on atherosclerotic ischemic stroke (IS) risk.

**Patients and methods:**

A case-control study was conducted. A total of 360 patients with atherosclerotic IS and 342 controls between December 2018 and December 2019 from Longyan First Hospital affiliated to Fujian Medical University were included. A logistic regression model was used to examine the association between SNPs and atherosclerotic IS risk. Odds ratios (ORs) and 95% confidence intervals (95% CIs) were calculated. Generalized multifactor dimensionality reduction was employed to analyze the SNP-SNP interaction.

**Results:**

Logistic regression analysis showed that atherosclerotic IS risk was significantly lower in carriers with the rs11672433-T allele than those with the CC genotype (CT+ TT vs. CC); adjusted OR, 0.005; 95% CI, 0.02–0.11. We found a significant 2-locus model (*P* = 0.0010) involving rs11672433 and rs4076317; the cross-validation consistency of this model was 10 of 10, and the testing accuracy was 57.96%. Participants with the CT or TT of rs11672433 and CC of rs4076317 genotype have the lowest atherosclerotic IS risk, compared to subjects with CC of rs11672433 and the CC of rs4076317 genotype, OR (95%CI) was 0.06(0.02–0.22), after covariates adjustment for gender, age, smoking and alcohol status, hypertension, Diabetes mellitus, TG, TC, HDL-C, LDL-C, Uric acid.

**Conclusions:**

We found that rs11672433 was associated with decreased atherosclerotic IS risk; we also found that gene–gene interaction between rs11672433 and rs4076317 was associated with decreased atherosclerotic IS risk.

## Introduction

Angiopoietin-like protein 4 (ANGTPL4) is a secreted glycoprotein that has been shown to regulate angiogenesis and to be involved in lipid, glucose, energy metabolism, wound healing, tumorigenesis, and redox regulation [1] .The human ANGPTL4 gene is located on chromosome 19p 13.3, which has seven exons and six introns, encoding a 406-amino-acid glycoprotein with a molecular mass of 45–65 kD [2]. ANGPTL4 forms oligomers and is found in glycosylated and cleaved isoforms [3]. ANGPTL4 contains a N-terminal coiled-coil domain and a large, C-terminal fibrinogen-like domain, and different domains of ANGPTL4 may have distinct physiological functions [4, 5]. Lipoprotein lipase (LPL) plays an important role in regulating plasma triglyceride levels [6]. The main function of LPL is to catalyze the hydrolysis of triglycerides (TG) in plasma TG-rich lipoproteins such as chylomicrons (CM) and very low-density lipoproteins (VLDL) into glycerol and fatty acids (FFAs) [7]. In general, LPL is viewed as an anti-atherogenic enzyme because of its action on lipoproteins [8, 9]. ANGPTL4 inhibits the activity of LPL, thus suppressing the clearance of circulating triglycerides [10]. It has been reported that Angptl4-deficient mice have better lipid metabolism and smaller atherosclerotic lesions than mice without this deficiency [11, 12]. ANGPTL4 influences the lipid metabolism thus involved in the pathogenesis of the chronic inflammatory process of atherosclerosis. Therefore, it is reasonable to believe that ANGPTL4 plays an important role in the occurrence and development of human atherosclerosis [13]. Atherosclerosis is an inflammatory disease that can lead to several complications such as ischemic stroke, heart disease, and peripheral vascular disease [14]. According to data from the 2012 NHANES (National Health and Nutrition Examination Survey), overall stroke prevalence is an estimated 2.6% in the American population [15]. Studies have shown that atherosclerosis IS the most common cause of cerebral infarction, accounting for 37.3% of cases [16]. In addition, most of genetic polymorphisms of ANGPTL4 genes were investigated in relation to the energy metabolism and coronary artery disease [17–20]. To date, there are very few studies on the association of ANGPTL4 gene polymorphisms with atherosclerotic IS susceptibility in humans. Additionally, multiple genotypes, such as ANGPTL4, can be the result of many single nucleotide polymorphisms (SNPs) and multiple SNP-SNP interactions. Until now, no study has focused on the impact of SNP interactions within ANGPTL4 gene polymorphisms on atherosclerotic IS in humans.

Thus, the aim of this study was to investigate the association of several SNPs in the ANGPTL4 gene, and SNP-SNP interactions on susceptibility to atherosclerotic IS based on a Chinese population.

## Materials and methods

### Study population

Our study was approved by the ethics committees of the Longyan First Hospital affiliated to Fujian Medical University, in compliance with the Declaration of Helsinki. Each of the participants provided written, informed consent before participating in this study.

A case-control study was conducted and a total of 360 patients with atherosclerotic IS and 342 controls were included. All participants were recruited between December 2018 and December 2019 from Longyan First Hospital affiliated to Fujian Medical University. Ischemic stroke was confirmed based on both clinical findings and the results of brain magnetic resonance imaging (MRI). Ischemic stroke in all cases was due to atherothrombotic (AT), according to the Trial of ORG 10172 in the Acute Stroke Treatment (TOAST) classification system [21]. Exclusion criteria included: (1) family history of apoplexy or a previous history of strokes; (2) cardiogenic cerebral embolisms or ischemic stroke caused by unknown factors; (3) cerebral hemorrhage, serious cardiac disease, severe liver disease, renal failure, hematologic or autoimmune diseases, chronic inflammatory diseases; (4) inability to undergo MRI imaging; (5) individuals who declined to participate in the study. The controls were recruited from patients who received physical examination in hospitals during the same period without any atherosclerotic diseases and stroke history, as confirmed by medical history.

Data on various risk factors were recorded, including age, gender, smoking, and drinking information, history of diabetes mellitus and hypertension. Fasting blood samples were tested for blood sugar, triglycerides (TG), total plasma cholesterol (TC), high-density lipoprotein cholesterol (HDL-C), low-density lipoprotein cholesterol (LDL-C), and uric acid.

### Genotyping of SNPs

Owing to the limitations of manpower, material, and financial resources, a total of 3 potentially functional SNPs within the ANGPTL4 gene were selected for genotyping in this study: rs11672433, rs4076317, and rs1044250. The SNPs were selected based on the NCBI database (http://www.ncbi.nlm.nih.gov/projects/SNP) according to the following three criteria: 1) located in a gene fragment that could have functional effects; 2) MAF more than 5%; 3) previously reported associations with atherosclerosis, but were not well studied [18, 20]. Whole blood (5 ml) was drawn from an arm vein into a sterile tube containing ethylene diamine tetraacetic acid (EDTA) and stored at − 80 °C until genotype analysis was performed. SNP genotyping was performed by a matrix-assisted laser desorption/ionization time-of-flight mass spectrometry (MALDI-TOF MS) method. Genotyping was performed in real time with Mass ARRAY RT software version 3.0.0.4 and analyzed using Mass ARRAY Typer software, version 3.4 (Sequenom Inc., San Diego, CA, USA).

### Statistical analysis

Statistical analyses were performed by using the IBM SPSS Statistics 20.0 software (IBM Corporation, NY, USA). The means and standard deviations (SDs) were calculated for normally distributed continuous variables, and percentages were calculated for categorical variables. Categorical data were compared using the χ^2^-test, and continuous variables were compared using the Student t-test. Hardy–Weinberg equilibrium was analyzed by χ^2^-test. SNP-SNP interaction was assessed using the generalized multifactor dimensionality reduction (GMDR) method. Some parameters, including cross-validation consistency, the testing balanced accuracy, and the sign test, to assess each selected interaction were calculated.

## Results

### Demographic and clinical characteristics of the study population

A total of 702 participants were enrolled in the present study, including 360 atherosclerotic IS patients and 342 control participants. The demographic and clinical characteristics of atherosclerotic IS patients and control participants are shown in Table 1. The mean age, number of smokers, drinkers, levels of triglycerides (TG), high-density lipoprotein cholesterol (HDL-C) and uric acid were not significantly different between patient and control populations (*P*>0.05). There was a significantly higher proportion of males in the patient population, and rates of hypertension, diabetes mellitus, fasting blood-glucose (FBG), total cholesterol (TC), and low-density lipoprotein cholesterol (LDL-C) were higher in the case group (*P*<0.05).

**Table 1:** General characteristics of 702 study participants in case and control group. Data presented as mean ± SD

Variables	Case Group *n* = 360	Control Group *n* = 342	*P* values
Men, N (%)	236 (65.6)	181 (52.9)	0.001
Mean age, N (years)	66.47 ± 12.34	65.33 ± 8.20	0.149
Smoke, N (%)	134 (37.2)	112 (32.7)	0.214
Alcohol consumption, N (%)	78 (21.7)	78 (22.8)	0.716
Diabetes mellitus, N (%)	122 (33.9)	53 (15.5)	0.000
FBG (mmol/L)	7.15 ± 3.30	5.98 ± 1.70	0.000
Hypertension, N (%)	271 (75.3)	165 (14.1)	0.000
TG (mmol/L)	1.84 ± 1.81	1.76 ± 1.36	0.507
TC (mmol/L)	5.13 ± 1.22	4.71 ± 1.09	0.000
HDL-C (mmol/L)	1.37 ± 0.49	1.39 ± 0.49	0.680
LDL-C (mmol/L)	3.27 ± 0.98	2.95 ± 0.99	0.000
Uric acid (mmol/L)	353.66 ± 115.04	351.72 ± 94.53	0.807

Abbreviations: *FBG* fasting blood-glucose; *TG* triglycerides; *TC* total cholesterol; *HDL-C* high-density lipoprotein cholesterol; *LDL-C* low-density lipoprotein cholesterol

### Association of the ANGPTL4 SNPs with the risk of atherosclerotic ischemic stroke

The genotypic and allelic frequencies of the ANGPTL4 SNPs are presented in Table 2. No significant difference in genotype frequencies from the Hardy-Weinberg equilibrium (HWE) test was noted for any tested SNP in the control cases (*P* > 0.05). The frequency for the rs11672433-T allele was significantly lower in cases than controls (2.5% vs. 10.4%). Logistic regression analysis showed that stroke risk was significantly lower in carriers with the rs11672433-T allele than those with the CC genotype (CT+ TT vs. CC: adjusted OR, 0.005; 95% CI, 0.02–0.11). However, we did not find any significant association between rs4076317, rs1044250 and stroke after covariate adjustment.

**Table 2:** Genotype and allele frequencies of three SNPs between case and control group

SNP	Genotypes and alleles	Frequencies N (%)		OR (95% CI)^a^	*P*-value	HWE test for case	HWE test for controls
		Cases *n* = 360	Control *n* = 342				
rs11672433							
Genotype	CC	348 (96.7)	278 (81.3)	1.00		0.000	0.054
	CT	6 (1.7)	57 (16.7)	0.15 (0.06–0.37)	0.000		
	TT	6 (1.7)	7 (2.0)	0.09 (0.02–0.52)	0.007		
	CT + TT	12 (3.3)	64 (18.8)	0.05 (0.02–0.11)	0.000		
Allele	C	702 (97.5)	613 (89.6)	0.22 (0.13–0.38)	0.000^b^		
	T	18 (2.5)	71 (10.4)				
rs4076317							
Genotype	CC	179 (49.7)	154 (45.0)	1.00		0.633	0.402
	CG	147 (40.8)	156 (45.6)	0.84 (0.58–1.20)	0.330		
	GG	34 (9.4)	32 (9.4)	0.87 (0.47–1.61)	0.662		
	CG + GG	181 (50.3)	188 (55.0)	0.83 (0.58–1.19)	0.317		
Allele	C	505 (70.1)	464 (67.8)	0.90 (0.72–1.13)	0.351 ^b^		
	G	215 (29.9)	220 (32.2)				
rs1044250							
Genotype	CC	315 (87.5)	292 (85.4)	1.00		0.001	0.165
	CT	39 (10.8)	46 (13.4)	1.07 (0.60–1.90)	0.814		
	TT	6 (1.7)	4 (1.2)	2.82 (0.41–4.92)	0.291		
	CT + TT	45 (12.5)	50 (14.6)	1.01 (0.62–1.66)	0.758		
Allele	C	669 (92.9)	630 (92.1)	0.89 (0.60–1.32)	0.563 ^b^		
	T	51 (7.1)	54 (7.9)				

^a^Adjusted for gender, age, smoking and alcohol status, hypertension, diabetes mellitus, TG,TC, HDL-C, LDL-C, Uric acid

^b^ Chi-square test for the allele frequencies between the cases and controls

### SNP–SNP interactions

The GMDR model was used to screen the best interaction combinations among 3 SNPs. The results of the analysis of the SNP–SNP interactions between the three SNPs and the risk of atherosclerotic IS are summarized in Table 3. We found that there was a significant two-locus model (*P* = 0.001) involving rs11672433 and rs4076317, indicating a potential gene–gene interaction between rs11672433 and rs4076317. Overall, the cross-validation consistency was 10/10, and the testing accuracy was 57.96%. To evaluate the joint effect of rs11672433 and rs4076317 on atherosclerotic IS risk, we conducted stratified analysis between the 2 SNPs by using logistic regression. We found that subjects with CT or TT of rs11672433 and CC of rs4076317 genotype have the lowest atherosclerotic IS risk, compared to subjects with CC of rs11672433 and the CC of rs4076317 genotype, OR (95%CI) was 0.06(0.02–0.22), after adjustment for gender, age, smoking and alcohol status, hypertension, diabetes mellitus, TG, TC, HDL-C, LDL-C, Uric acid **(**Table 4**).**

**Table 3:** GMDR Analysis on the Best SNP-SNP Interaction Combinations

Locus no.	Best combination	Testing accuracy	Cross Validation consistency	*P*-value^a^
2	rs11672433, rs4076317	0.5796	10/10	0.0010
3	rs11672433, rs4076317, rs1044250	0.5870	10/10	0.0647

*P*-values less than 0.05 were considered statistically significant. Abbreviations: *GMDR* generalized multifactor dimensionality reduction; *SNP* single-nucleotide polymorphism

^a^ Adjusted for gender, age, smoking and alcohol status, hypertension, diabetes mellitus, TG, TC, HDL-C, LDL-C, Uric acid

**Table 4:** Interaction analysis for rs11672433 and rs4076317 by using logistic regression

rs11672433	rs4076317	OR (95%CI)	*P*-value^a^
CC	CC	1.0	–
CT or TT	CC	0.06 (0.02–0.22)	< 0.001
CC	CG or GG	0.74 (0.52–1.06)	0.032
CT or TT	CG or GG	0.24 (0.10–0.54)	0.482

^a^ Adjusted for gender, age, smoking and alcohol status, hypertension, diabetes mellitus, TG, TC, HDL-C, LDL-C, Uric acid

## Discussion

Lipid metabolism disorder is the pathological basis of atherosclerosis [22]. Lipid metabolism is a complex physiological process, which involves the cooperation of many proteins. Lipoprotein lipase (LPL) hydrolyzes fatty acids (FAs) from triglyceride (TAG)-rich lipoproteins, including very low-density lipoproteins (VLDLs) and chylomicrons, and regulates their distribution to peripheral tissues [23]. Angiopoietin-like protein 4 (Angptl4) is an endogenous inhibitor of the triglyceride-hydrolyzing enzyme lipoprotein lipase (LPL) that catalyzes uptake of circulating lipids into tissues [11, 24]. ANGPTL4 influences a series of actions implicated in atherosclerosis (AS), including inflammation and lipid accumulation [11, 12]. Accumulating evidence associates the serum levels and gene polymorphisms of ANGPTL4 directly with the risk of atherosclerosis [23, 25, 26].

In recent years, several studies have attempted to address the association between the ANGPTL4 SNPs and arterial disease in humans. In the current study, we investigated the impact of several SNPs in the ANGPTL4 gene on atherosclerotic IS risk. We found that the rs11672433 variant was related to human susceptibility to atherosclerotic IS. The SNP rs11672433 is located in the C-terminal region of ANGPTL4 [27]. However, Guo et al. [28] found that the C-terminus of ANGPTL4 represents a master regulator of vascular permeability and angiogenesis in endothelial cells. Thus, it may be assumed that rs11672433 may influence atherosclerotic IS risk by adjusting the activity of ANGPTL4 to reduce angiogenesis (leading to plaque destabilization and rupture) and vascular permeability. In agreement with the findings of Qian Yang et al. [29], we found no significant correlation of rs4076317 SNPs with atherosclerotic IS susceptibility. This is in contradiction to the findings of Xin-Wei He et al. [25], which indicated that rs4076317 SNPs in the ANGPTL4 gene was associated with atherosclerotic IS, they found that a lower proportion of LAA stroke cases (7.0%) were homozygous for the minor allele (G) of rs4076317 compared with the controls (10.9%). The rs4076317 polymorphism is located within 5′ N-terminal region (the promoter region) of the ANGPTL4 gene [30]. The N-terminal and full-length ANGPTL4 in the blood stream inhibited the activity of blood LPL [28]. Studies on rs4076317 in humans are scarce, and it remains unknown whether rs4076317 influences the N-terminal domain of ANGPTL4. We also found no significant correlation between rs1044250 SNPs and atherosclerotic IS susceptibility, consistent with previous findings [20]. Interestingly, Kaouthar Abid et al. [17] concluded that the rs1044250 SNPs was associated with coronary artery disease risk in T2D Tunisian patients. To determine whether rs1044250 has an effect on the risk of atherosclerotic IS in the general population requires further studies with larger sample sizes.

Many important genes associated with atherosclerotic IS have been reported previously [31–34], mostly identified based on single-SNP disease models which do not address the potential joint effects of multi-loci SNPs. However, it was reported that multiple non-significant SNPs can generate joint effects that are associated with diseases [35]. Thus, it was necessary and interesting to investigate the impact of SNP-SNP interaction on atherosclerotic IS risk. We found a significant SNP-SNP interaction between rs11672433 and rs4076317. Participants with the CT or TT of rs11672433 and the CC of rs4076317 genotype have the lowest atherosclerotic IS risk, compared to patients with the CC of rs11672433 and CC of rs4076317 genotype. To our knowledge, this was the first study to investigate ANGPTL4 gene SNP-SNP interaction on the risk of atherosclerotic IS in a Chinese population. The detailed mechanism for this interaction is not well-known; however, we believe that correlations and interactions among rs11672433 and rs4076317 may play critical roles in the development of atherosclerotic IS.

The potential limitations of our study warrant consideration. First, only 3 SNPs within the ANGPTL4 gene were chosen for this study. Additional SNPs should be included in further studies. Second, gene environment interaction should be investigated in future studies. Third, the current study is limited to a single nationality and should be validated in future studies with a larger sample size that includes different nationalities.

In conclusion, our findings support the existence of an association between rs11672433 in the ANGPTL4 gene and the risk of developing atherosclerotic IS in a Chinese Han population. In particular, individuals carrying the rs11672433 T allele and rs4076317 C allele of the ANGPTL4 gene may have a lower risk of developing atherosclerotic IS. The results may provide clues for the evaluation of individual susceptibility to atherosclerotic IS and may serve in the development of effective prevention strategies.
